# Platelet endothelial aggregation receptor-1 regulates bovine muscle satellite cell migration and differentiation via integrin beta-1 and focal adhesion kinase

**DOI:** 10.1080/19336918.2019.1619434

**Published:** 2019-05-27

**Authors:** Yusheng Pang, Ziheng Zhang, Zhao Wang, Yuxin Wang, Yunqin Yan, Shufeng Li, Huili Tong

**Affiliations:** The Laboratory of Cell and Developmental Biology, Northeast Agricultural University, Harbin, Heilongjiang, China

**Keywords:** PEAR1, integrin beta-1, focal adhesion kinase, migration, differentiation

## Abstract

PEAR1 is highly expressed at bovine MDSC differentiation. However, its biological function remains unclear. Western blotting results showed that PEAR1 increased between day 0 and day 2 of cell differentiation and decreased from day 3. Moreover, scratch test showed that wound healing rate increased after PEAR1 overexpression and decreased upon its suppression. Meanwhile, we found that, upon PEAR1 induction, both the expression of the focal adhesion-associated and MyoG, and the myotube fusion rate increased. However, when PEAR1 was suppressed, opposite results were obtained. Immunoprecipitation revealed an association between PEAR1 and ITGB1. Notably, inhibition of FAK and ITGB1 repressed cell differentiation. In conclusion, our study indicated that PEAR1 is involved in the regulation of bovine MDSC migration and differentiation.

## Introduction

Platelet endothelial aggregation receptor-1 (PEAR1;also known as JEDI or MEGF12) is a type-1 transmembrane protein of the multiple epidermal growth factor – like domain protein family. PEAR1 is composed of an extracellular EMI domain (protein-protein interaction domain), including 15 extracellular EGF-like repeats, and an intracellular domain containing multiple cytoplasmic tyrosines,5 proline-rich domains, and an NPXY922 motif possibly serving as a phosphotyrosine binding site [1]. PEAR1 is mainly expressed in platelets and endothelial cells, as well as satellite glial cell precursors [2]. DxS promotes platelet aggrega – tion by directly phosphorylating PEAR1 to activate PI3K/Akt [3]. During development of the peripheral nervous system, PEAR1 promotes phagocytosis through a non-classical phosphor – ylation-dependent mechanism [4]. In cultured endothelial cell, knockdown of PEAR1 doubled the proliferation rate and significantly stimulated cell migration, which enhanced *in vitro* tube formation on matrigel through the Akt/PTEN-dependent p21/CDC2 pathway. In addition, overexpression of PEAR1 in NIH 3T3 fibroblasts resulted in a reduction of late and early myeloid progenitors in non-adherent co-cultured bone marrow cells, suggesting that PEAR1 is involved in the early stages of hematopoietic differentiation [5].

Cell migration is a coordinated process that involves rapid changes in the dynamics of actin filaments, together with the formation and disassembly of cell adhesion sites. The extracellular matrix, integrins, and the cellular cytoskeleton interact at the cell junctions to form focal adhesions, affecting cell migration and adhesion, as well as signal transduction [6]. The intracellular domain of integrins binds to the cytoskeleton via adapter proteins such as talin, α-actinin, vinculin, focal adhesion kinase, and paxillin [7]. Focal adhesion kinase (FAK) is a unique molecular linker functioning as an important receptor proximal regulator of cell shape, adhesion, and motility [8]. In the migration of retinal pigment epithelial cells, FAK regulates cell transformation and migration by tyrosine phosphorylation [9]. FAK could participate in BMP-9 induced ADSCs osteogenesis via the Wnt signaling pathway [10]. Among the many membrane receptors that can mediate the communication between the ECM and migrating cells, the integrin mediated focal adhesion (FA) is one of the most important [11]. During plasma cell differentiation, a reduced activation of β1-integrin contributes to the impaired plasmablast differentiation and migration of antibody-secreting cells to bone marrow [12]. Down regulation of integrin β1 severely affects osteoblast differentiation [13]. During skeletal muscle development, myoblast fusion is fundamental to the development and regeneration of skeletal muscles. In order to fuse, MDSCs must undergo cell-cell recognition and adhesion and merger of membranes between apposing cells. Cell migration must occur prior to these events to bring myoblasts into proximity [14]. In mice, knocking out genes necessary for satellite cell migration leads to blockage of muscle regeneration [15].

Although a relationship between PEAR1 and cell migration has been reported, the relevant molecular mechanisms are still unclear. PEAR1 has been reported to PEAR1 promote C2C12 cells differentiation and the repair of mouse skeletal muscle damage, but the molecular mechanism is also still unclear [16]. Deep Sequencing has shown that PEAR1 RNA expression is highly at early stages of bovine MDSC differentiation. However, the function and expression pattern for PEAR1 in bovine MDSCs has not been reported [17]. In this study, we aimed to explore the role of PEAR1 in bovine MDSC differentiation and its role in cell migration. Our work may provide a theoretical basis for understanding the mechanisms of bovine skeletal muscle development.

## Results

### PEAR1 expression during bovine MDSC differentiation

**Figure 1.**
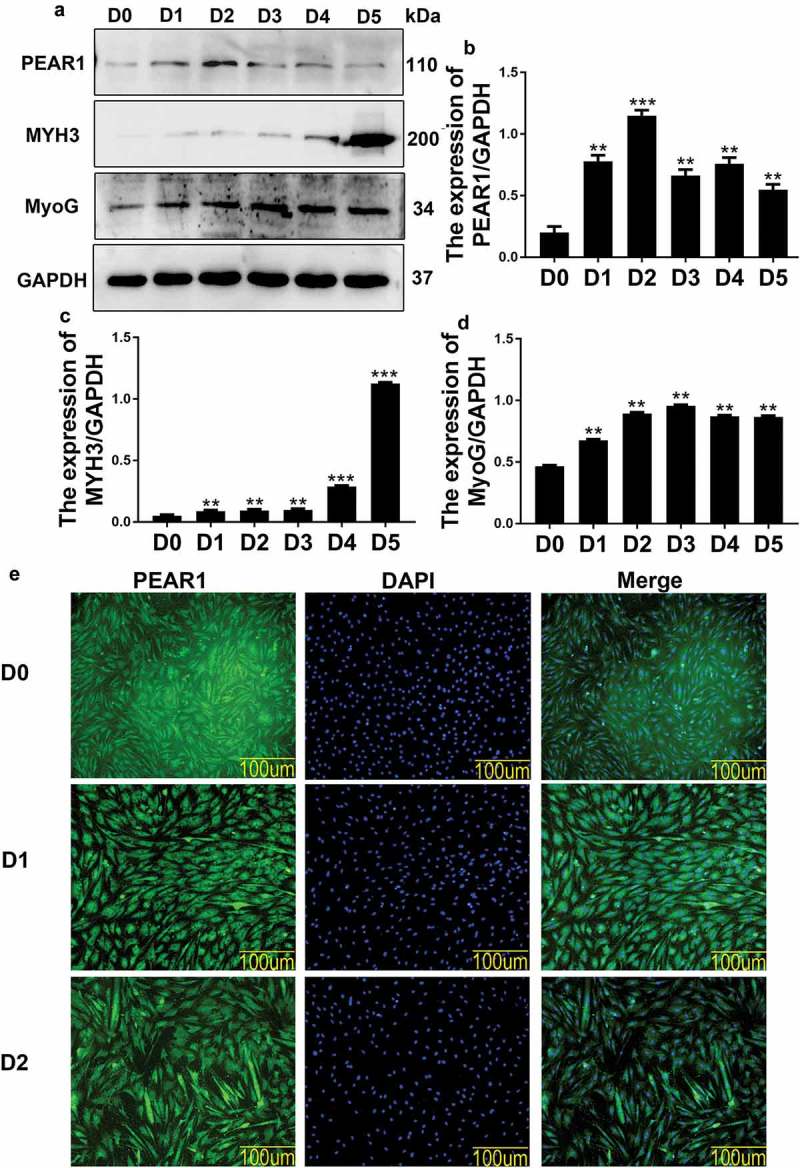
PEAR1 expression during bovine MDSC differentiation. (A) Western blotting analysis of differentiated MDSCs, showing the expression of PEAR1, MYH3, and MyoG at various stages. D0 indicates undifferentiated MDSCs and D1 to D5 indicate MDSCs on days 1 to 5, respectively, after initial exposure to differentiation medium. (B-D) Quantification of PEAR1, MYH3, and MyoG protein expression at various differentiation stages; compared to the D0 group (n = 3), *P < 0.05, **P < 0.01, **P < 0.001, NS: no significant difference. (E) Immunofluorescent staining for PEAR1 in MSDCs on differentiation day 0 (D0), 1 (D1), and 2 (D2). The green and blue signals represent PEAR1 and DAPI-stained nuclei, respectively (magnification, 200×).

Western blotting and immunofluorescence analyses were used to determine PEAR1 protein expression and cellular localization at various differentiation stages of bovine MDSCs. PEAR1 protein levels were analyzed on days 0, 1, 2, 3, 4, and 5 of cell differentiation (Figure 1). The expression of PEAR1 increased from day 0 to day 2 and then gradually decreased from the third day (Figure 1(a,b)). The MYH3 protein level continuously increased during the differentiation period (Figure 1(a,c)). The expression of MyoG gradually increased from day 0 to day 3 and then decreased (Figure 1(a,d)).

Immunofluorescence results showed the presence of PEAR1 signal during bovine MDSC differentiation (Figure 1(e)).

### PEAR1 affects cell migration and interacts with ITGB1

**Figure 2.**
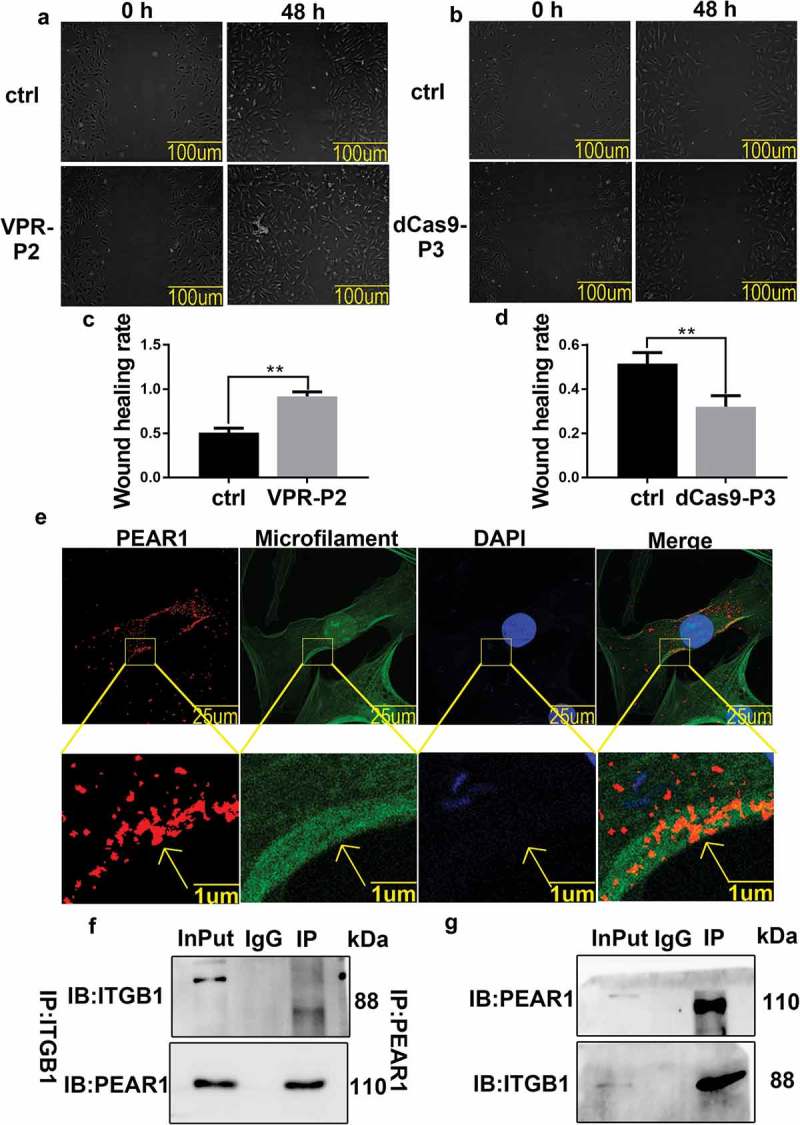
PEAR1 affects cell migration and interacts with ITGB1. (A-B) Cell scratch test. Ctrl is a sample transfected with the empty vector, VPR-P2 is a sample transfected with PEAR1 overexpression vector, dCas9-P3 is the suppression vector; ‘0 h’ indicates cells before migration, and ‘48 h’ indicates cell migration for 48 h under differentiation conditions. (C-D) Quantification of cell migration assay after overexpression or suppression of PEAR1. **P < 0.01, compared to the 0 h group (n = 3). (E) Immunofluorescent staining for PEAR1, microfilaments, and nuclei under differentiation conditions. The red signal represents PEAR1, the green signal represents microfilaments, and the blue signal represents nuclei stained with DAPI. (F) Immunoprecipitation with anti-PEAR1 antibodies followed by western blotting with anti-ITGB1 antibodies. InPut is a positive control, IgG is a negative control, IP is the target. (G) Immunoprecipitation with anti-ITGB1 antibodies followed by western blotting with anti-PEAR1 antibodies.

CRISPR is an effective tool for targeted gene editing in many organisms. We applied CRISPR system, using the designed vectors VPR-sgRNA(VPR-P2),and dCas9-sgRNA (dCas9-P3) for PEAR1 overexpression or silencing,respectively. We used transient transfection and examined the effect of these manipulations on cell migration during differentiation (Figure 2(a,b)). The scratch test showed that the wound healing rate increased after PEAR1 overexpression and decreased after PEAR1 suppression (Figure 2(a-d)). We also explored the intracellular localization of PEAR1 and microfilaments by immunofluorescence (Figure 2(e)), showing that PEAR1 was localized on the cell membrane and in the cytoplasm. Notably, PEAR1 and microfilament staining patterns were partially overlapped.

To further explore the mechanism by which PEAR1 affects cell differentiation, we performed immunoprecipitation and QE mass spectrometry analysis (Supporting Information Table 1). From the results of mass spectrometry identification, we speculate that PEAR1 may regulate cell migration and differentiation by binding to integrin beta1(ITGB1). Therefore, we performed co-immunoprecipitation experiments to verify this interaction, using either anti-ITGB1 antibodies for immunoprecipitation, followed by western blotting with anti-PEAR1 antibodies (Figure 2(f)), or vice versa (Figure 2(g)). In both cases, we detected an interaction between the two proteins.

### PEAR1 affected focal adhesions and bovine MDSC differentiation

**Figure 3.**
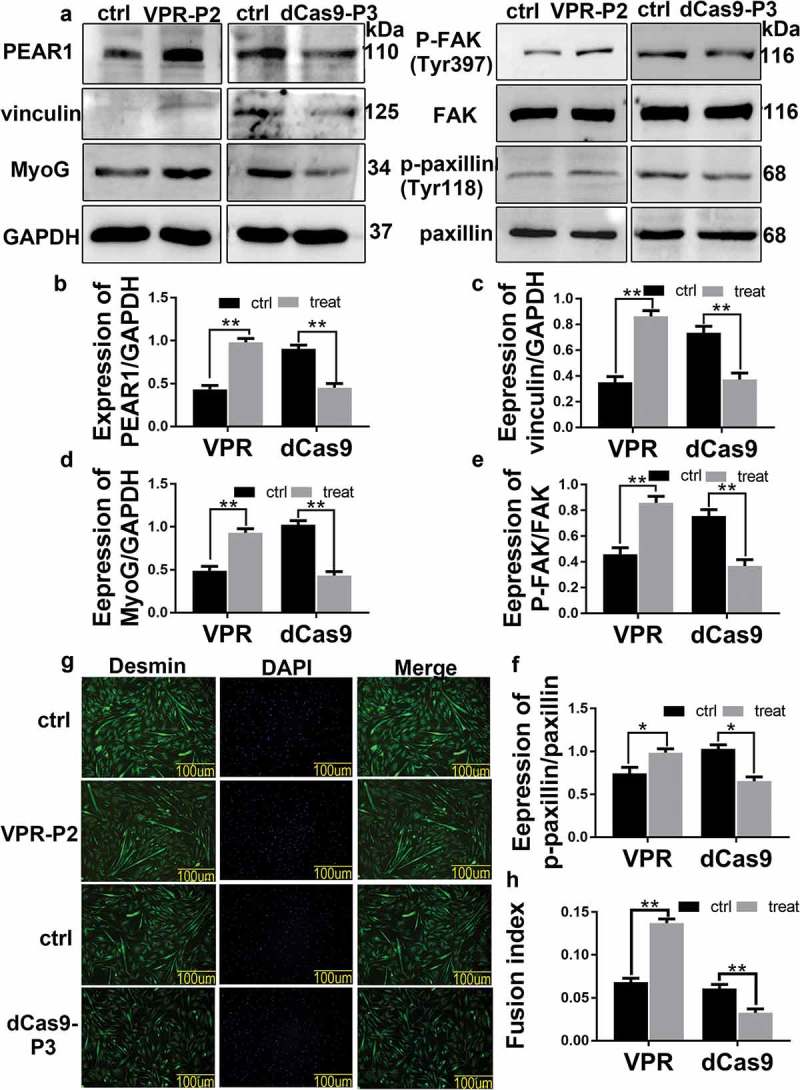
PEAR1 affected the focal adhesions and bovine MDSC differentiation. (A) PEAR1 overexpression or suppression in bovine MDSCs. Ctrl is a sample transfected with the empty vector, VPR-P2 is a sample transfected with PEAR1 overexpression vector, dCas9-P3 is the suppression vector; (B-F) Quantification of the results shown in A. (G) Immunofluorescentce staining of Desmin in bovine MDSCs following PEAR1 overexpression or interference (magnification, 200×). (H) Quantification of myotube fusion rate based on desmin staining and visualization of total nuclei with DAPI. *P < 0.05, **P < 0.01, **P < 0.001, NS: no significant difference, compared to the control group (n = 3).

We used western blotting to verify PEAR1 expression after transient transfection, and examine the impact of PEAR1 upregulation or downregulation on the expression of the focal adhesion related proteins, p-FAK, p-paxillin, and vinculin, and the differentiation marker, MyoG (Figure 3). As expected, PEAR1 expression was increased after transfection of the overexpression vector (VPR-P2) and detected after transfection of the silencing vector(dCas9-P3) (Figure 3(a,b)). The expression of p-FAK,p-paxillin,vinculin,MyoG was found to increase after PEAR1 overexpression, whereas it decreased after PEAR1 suppression (Figure 3(a,c-f)). Desmin is specifically expressed in skeletal muscles. Thus, we used it as a marker of bovine MDSC differentiation and determined its localization by immunofluorescence(Figure 3(g)). Image analysis showed that the myotube fusion rate was enhanced after PEAR1 overexpression (Figure 3(g,h)) but decreased after PEAR1 suppression, compared to control cells (Figure 3(g,h)).

### FAK inhibition affects bovine MDSC differentiation

**Figure 4.**
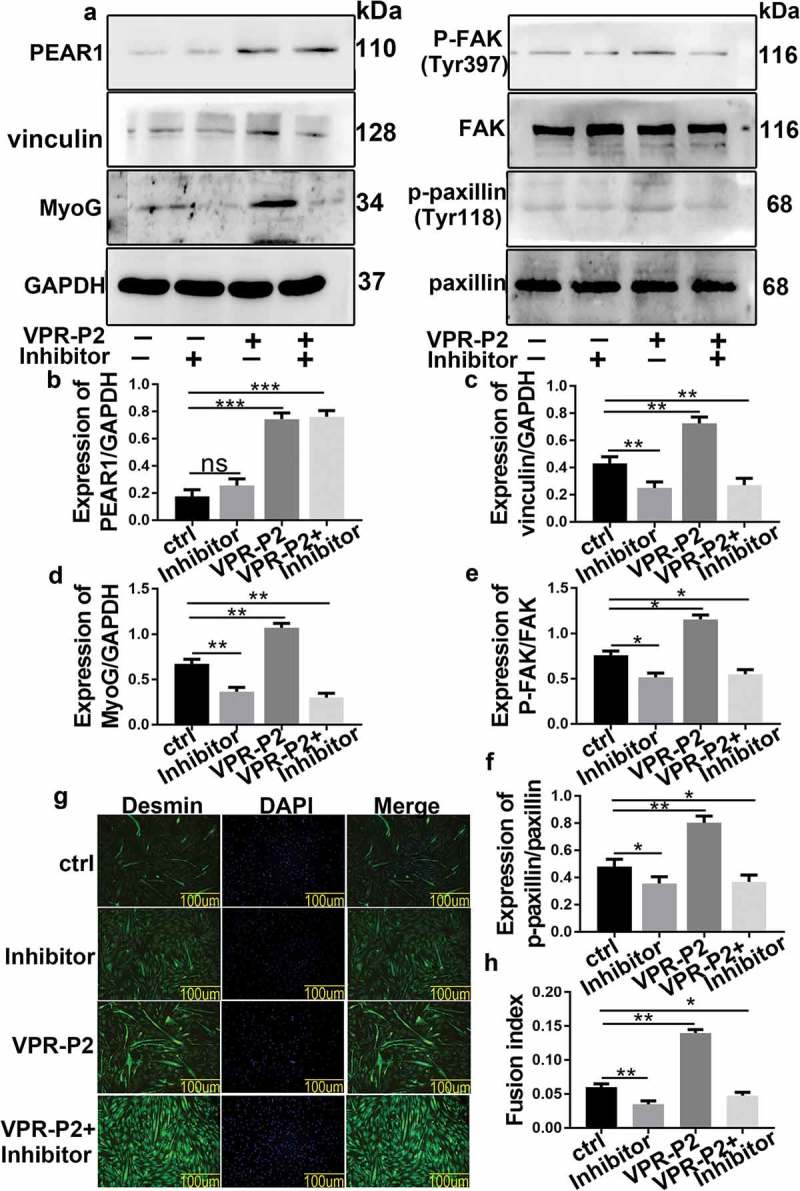
FAK inhibition affects bovine MDSC differentiation. (A) Inhibits FAK activity, overexpresses PEAR1, overexpresses PEAR1 and inhibits FAK activity expression in bovine MDSCs for differentiation 48 h after treatment. (B-F) Quantification of the results shown in A . (G) Immunofluorescent staining for desmin in MDSCs following FAK inhibition, PEAR1 overexpression, and PEAR1 overexpression in the presence of the FAK inhibitor (magnification, 200×). (H) Quantification of the myotube fusion rate based on desmin staining and visualization of total nuclei with DAPI in G. *P < 0.05, **P < 0.01, **P < 0.001, NS: no significant difference.

The protein expression of MyoG, p-FAK, p-paxillin, and vinculin was investigated by western blotting upon suppression of FAK activity by a specific inhibitor (PF-562,271) (Figure 4). When PEAR1 expression was induced in the absence of FAK inhibitor, MyoG, p-FAK, p-paxillin, and vinculin expression was increased (Figure 4(a-f)). On the other hand, under conditions of FAK inhibition, PEAR1 overexpression failed to induce the expression of these proteins, whose level was lower compared to control cells (Figure 4(a-f)). Immunofluorescence staining showed that FAK inhibition resulted in a decrease in myotube fusion rate (Figure 4(g,h)). Similarly, although myotube fusion activity was increased by PEAR1 overexpression, FAK inhibition prevented this effect (Figure 4(g,h)).

### ITGB1 silencing affects bovine MDSC differentiation

**Figure 5.**
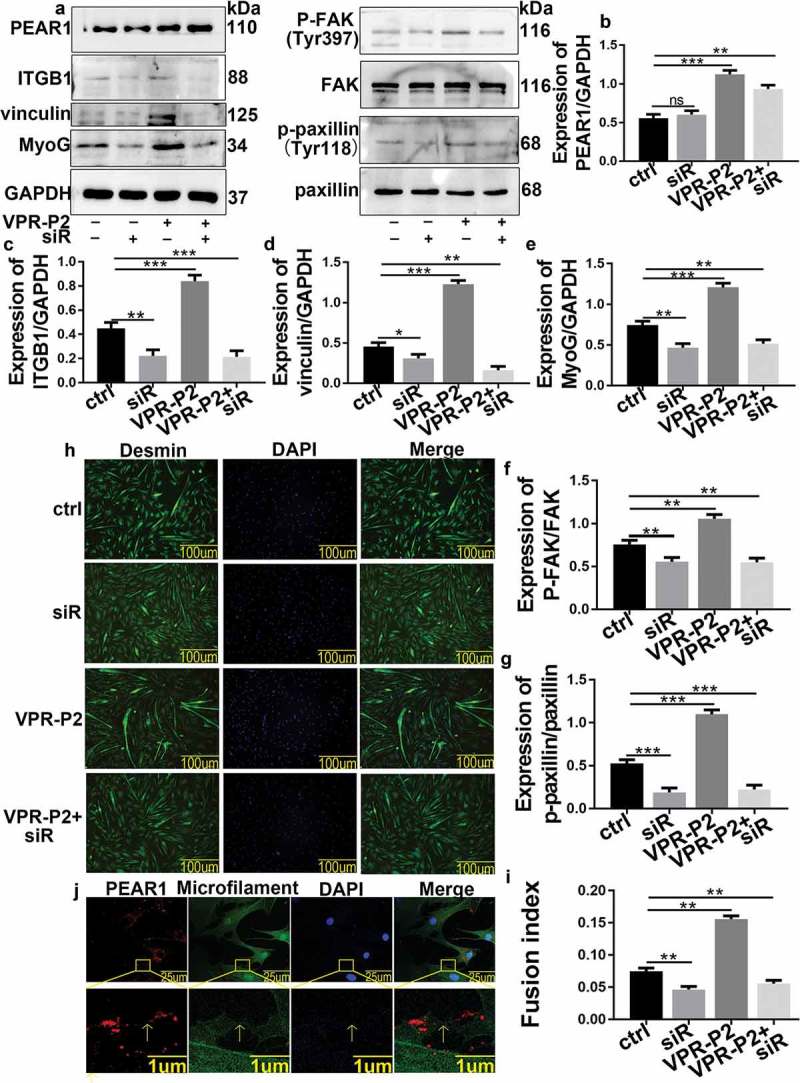
ITGB1 silence affects bovine MDSC differentiation. (A) ITGB1 suppression, PEAR1 overexpression, PEAR1 overexpression and ITGB1 suppression in MDSCs. (B-G) Quantification of the results shown in A. (H) Immunofluorescent staining of desmin in bovine MDSCs following ITGB1 suppression, PEAR1 overexpression, PEAR1 overexpression and ITGB1 suppression (magnification, 200×). (I) Quantification of myotube fusion rate based on desmin staining and quantification of total DNA (DAPI) from H. (J) Immunofluorescent staining for PEAR1, microfilaments, and nuclei in MSDCs on day 2 of differentiation. The red, green, and blue signals represent PEAR1, microfilament, and nuclei stained by DAPI, respectively. *P < 0.05, **P < 0.01, **P < 0.001, NS: no significant difference.

The protein expression of MyoG, p-FAK, p-paxillin, and vinculin was investigated upon ITGB1 silencing by a chemically synthesized siRNA(siR). Western blotting analysis showed that the expression of ITGB1, MyoG, p-FAK, p-paxillin, and vinculin decreased after silencing of ITGB1 (Figure5(a-g)). However, PEAR1 overexpression resulted in the increased expression of these proteins (Figure5(a-g)). Notably, PEAR1 induced ITGB1 expression was prevented by the simultaneous silence of ITGB1. As a result, under the latter conditions, the expression of MyoG, p-FAK, p-paxillin, and vinculin was also decreased, compared to control cells (Figure 5(a-g)). Immunofluorescence staining showed that the rate of myotube fusion, promoted by PEAR1 overexpression, decreased upon ITGB1 inhibition, which also prevented the promotion effects of PEAR1 (Figure 5(h,i)). We detected PEAR1-specific fluorescent staining where the cell adhesion (Figure 5(j)). Together, these results suggested that PEAR1-induced differentiation of bovine MDSCs depend on functional ITGB1.

## Discussion

In a previous study analyzing PEAR1 transcription during MDSC differentiation, we reported that PEAR1 expression gradually increased from day 0 to day 1 and began to decline on day 3 [17]. In differentiating C2C12 cells, PEAR1 protein level tended to increase and peaked on day 4 [16]. In bovine MDSCs, PEAR1 protein expression gradually increased from day 0 to day 2 and began to decline on day 3, in line with the results of our previous study. Although PEAR1 expression profile differed in the two cell lines, possibly reflecting differences between species, PEAR1 promoted the differentiation of both C2C12 and bovine MDSCs. Immunofluorescence assays showed that PEAR1 was expressed during bovine MDSC differentiation. Based on our results and literature reports, we speculate that PEAR1 may be involved in the differentiation of bovine MDSCs.

During skeletal muscle development, myoblasts migrate to the area of myofiber formation and differentiate into multinucleated myotubes [18]. Cell migration occurs in the early stage of differentiation, i.e., when PEAR1 expression is maximal. To explore the relationship between PEAR1 and cell migration during bovine MDSC differentiation, we employed PEAR1 overexpression or inhibition and then examined cell migration using a scratch test. We found that the rate of wound healing was enhanced and decreased by PEAR1 overexpression and silencing, respectively, indicating a role of PEAR1 as a promoter of cell migration,in line with others reports [2].Integrins, adaptor molecules (paxillin, focal adhesion kinase and vinculin), and microfilaments play key roles in the assembly of stable focal adhesions. Co-localization experiments revealed that the intracellular distribution of PEAR1 and microfilaments overlapped and PEAR1 staining tended to localize along microfilaments. This result further demonstrated that PEAR1 may be involved in cell migration. Therefore, we used western blotting to evaluate the impact of altered PEAR1 expression on the level of proteins involved in focal adhesion formation. We found that p-FAK, p-paxillin, and vinculin expression was increased after PEAR1 overexpression and decreased upon PEAR1 suppression, suggesting a role of PEAR1 in FA assembly. These experimental results indicate that PEAR1 may regulate cell migration by affecting FA assembly. Using QE analysis of proteins and immunosuppressive techniques, we found that PEAR1 might functionally interact with ITGB1, indicating a role of this interaction in FA assembly.

Differentiation of myoblasts is closely related to cell migration. Previous studies have pointed out that inhibition of myoblast migration leads to the formation of fewer and smaller myotubes [15–19].We used western blotting and immunofluorescence techniques to examine the effects of altered PEAR1 expression on cell differentiation. MyoG, as well as myotube fusion, increased after PEAR1 overexpression and decreased after PEAR1 suppression, indicating that PEAR1 promoted bovine MDSC differentiation. Taken together, we speculate that a high expression of PEAR1 in early stages of differentiation may accelerate focal adhesion assembly, and thereby, the migration of bovine MDSCs, bringing myoblasts into proximity and favoring cell differentiation.

Focal adhesion kinase (FAK) is an important receptor-proximal regulator of motility and tyrosine phosphorylation of FAK was associated with the formation of focal contacts [20]. Cell treatment with the FAK inhibitor, PF-562,271, caused a decrease in the expression of P-FAK, p-paxillin, and vinculin, indicating that FAK was essential in the formation of focal adhesions. This is consistent with a previous study [21]. Moreover, under the same conditions, the expression of MyoG and the rate of myotube fusion were decreased. This suggested that inhibition of focal adhesion impaired bovine MDSC differentiation. FAK inhibitors inhibited FA assembly,impairing MDSC migration,and ultimately inhibited MDSC differentiation.PEAR1 overexpression caused a rise in the level of p-FAK, indicating that PEAR1 may have a potential regulation on FAK activity. However, PEAR1 overexpression did not rescue FA assembly and cell differentiation in the presence of the FAK inhibitor. This further demonstrated that PEAR1 regulated cell migration, via FA, thereby affecting cell differentiation.

**Figure 6.**
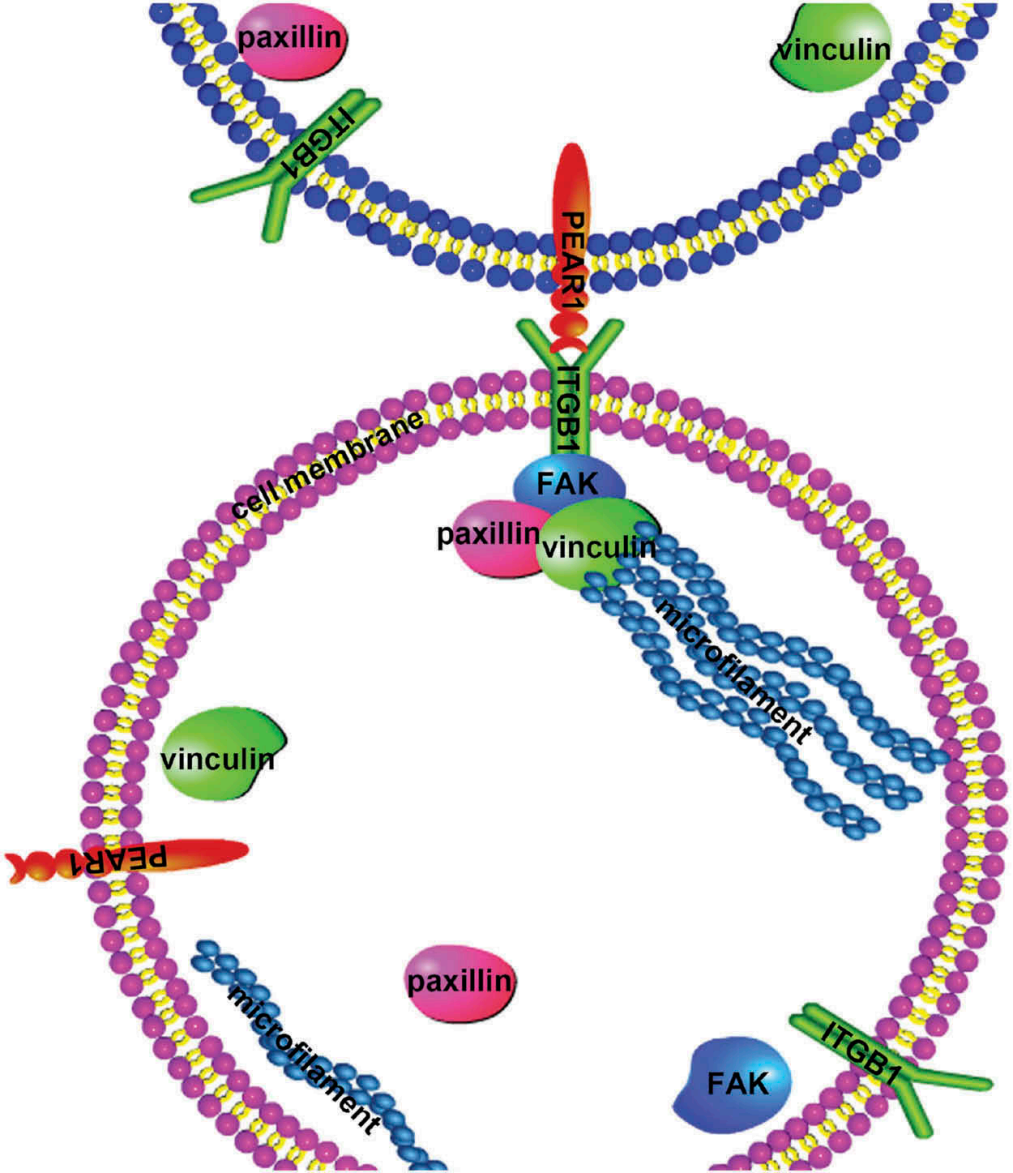
Scheme of PEAR1-mediated regulation of bovine MDSC migration and differentiation. In early stages of cell differentiation, the expression of PEAR1 increases at the cellular surface. Interactions between PEAR1 and its ligand, ITGB1, on adjacent cell membrane induce the formation of a complex. Activated ITGB1 receptor recruits focal adhesion proteins and a complex is formed, by FAK, paxillin, and vinculin, which in turn affects cell migration and differentiation.

ITGB1 is a transmembrane protein that controls cell migration by mediating dynamic interactions between the actin cytoskeleton and the extracellular matrix [22]. Given the possible association between PEAR1 and ITGB1, we used western blotting and immunization techniques to investigate whether PEAR1 could affect cell differentiation and migration through ITGB1. The results showed that ITGB1 inhibition resulted in decreased expression of p-FAK, p-paxillin, and vinculin, indicating that ITGB1was involved in the assembly of focal adhesions, influencing cell migration. MyoG and myotube fusion rates were also decreased under these conditions, suggesting that ITGB1 inhibition affected bovine MDSC differentiation. PEAR1 induction caused an increase in the expression of ITGB1, indicating a link between PEAR1 and ITGB1. Notably, under conditions of ITGB1 inhibition, the overexpression of PEAR1 was not able to rescue cell adhesion and differentiation. We found a PEAR1-specific signal on adjacent cell membranes, suggesting PEAR1 may be combined with ITGB1 on these cells. These results indicate that PEAR1 may affect the assembly of FA through ITGB1, which in turn affects bovine MDSC differentiation. During fibroblast migration, activated ITGB1 recruits focal adhesion kinase (FAK), which auto-phosphorylates Tyr-397 and subsequently recruits Src kinase [23].In summary, Our experimental results suggested that PEAR1 may be a new ligand for the ITGB1 receptor. In bovine MDSC differentiation, PEAR1 binds to ITGB1, and activation of ITGB1 affects FAK phosphorylation, and FA assembly, ultimately influencing bovine MDSC migration and differentiation (Figure 6).

In summary, PEAR1 was found to interact with ITGB1, affecting FA assembly, thereby influencing bovine MDSC migration and differentiation. The elucidation of the molecular mechanism may provide new insights into PEAR1 function in cell differentiation and migration.

## Materials and methods

### Cell culture and differentiation

Bovine MDSCs were isolated from the hind limb muscle tissue of newborn Chinese Simmental calves, according to a previously described method [24]. The protocol utilized in this study to harvest cells from animal tissues was approved by the Animal Care Commission of the Northeast Agricultural University and Heilongjiang, P.R. China. MDSCs were cultured in Dulbecco’s modified Eagle’s medium (DMEM, high glucose, Gibco) with 10% fetal bovine serum (Biological Industries), penicillin (100 units/ml) (Invitrogen), and streptomycin (60 ug/ml) (Invitrogen) at 37 °C in a 5% CO_2_ humidified atmosphere. During the differentiation of MDSCs, the growth medium was replaced by differentiation medium (2% horse serum (Biological Industries) in DMEM). The cells were transfected with plasmid DNA or siRNA (Sangon Biotech) by using Lipofectamine 2000 (Invitrogen) according to the manufacturer’s instructions.

### Overexpression and suppression of PEAR1 expression

To overexpress and suppress PEAR1 expression, we designed single guide RNAs (sgRNAs) targeting different sites of the PEAR1 promoter, and ligated them into the BbsI site of the pSPgRNA expression vector, which contains the hU6 promoter [25]. Bovine MDSCs in the logarithmic growth phase were seeded into 6-well plates. When cells reached 70–80% confluence, polyethylenimine (Sigma) was used for transfection. Cells were co-transfected with 2mg of the recombinant plasmid and 2mg of SP-dCas9-VPR plasmid (AddGene) or 2mg dCas9 plasmid (AddGene) for 48 h. Western blotting was used to detect PEAR1 expression levels, and screened for recombinant plasmids inducing the highest level of overexpression or suppression.

### ITGB1 silencing by RNA interference

Chemically synthesized siRNA was purchased from Sangon Biotech. Two interfering RNAs were synthesized. The siRNA sequences were designed as:

ITGB1_1: sense: GCGUACAAUUCCCUUUCUUTT, antisense: AAGAAAGGGAAU – UGUACGCTT. ITGB1_2: sense: GCUCAGGAAUGUUCACAUUTT, antisense: AAU-GUGAACAUUCCUGAGCTT

### FAK inhibitor treatment

PF-562,271 (MCE), a reversible inhibitor of FAK kinase, was used at 10 nM (in DMSO) and an equivalent volume of DMSO was used for the control treatments [28].

### Protein extraction and western blotting

MDSCs were lysed in RIPA buffer (Beyotime Biotechnology), according to the manufacturer’s instruction. The protein samples were resolved on an SDS-polyacrylamide gel and then transferred to a PVDF membrane (Millipore Corporation). The membrane was incubated with a primary antibody against PEAR1 (Abbkine), MYOG (Santa Cruz), GAPDH (Proteintech), MYH3 (Santa Cruz), p-FAK (Bioss), FAK(Bioss), p-paxillin (Bioss), paxillin (Bioss), vinculin (Bioss), or ITGB1 (Bioss), followed by addition of a secondary horseradish peroxidase (HRP)-labeled goat anti-rabbit IgG antibody (Bioss). The proteins were visualized by the Super ECL Plus detection kit (Applygen Technologies Inc) according to the manufacturer’s instructions. Images were acquired using the mini chemiluminescent imaging and analysis system MiniChemi™ 500 (Sage Creation Science).

### Immunofluorescence and co-localization

MDSCs were cultured sparsely on coverslips, washed three times with PBS, fixed in 4% paraformaldehyde at 20–22°C, and then permeabilized with 0.1% Triton X-100 in PBS(PBST). After blocking with 5% bovine serum albumin (BSA) in PBST. Cells were incubated with phalloidin, primary anti-PEAR1 or anti-desmin (Santa Cruz) antibodies, and then with FITC-conjugated goat anti-rabbit antibody. Coverslips were mounted with DAPI. Confocal images were visualized by laser scanning confocal microscopy or fluorescence mi-croscopy (Olympus). The myoblast fusion index was calculated via Image J soft-ware as the ratio between the number of nuclei inside myotubes and the total number of nuclei.

### Co-immunoprecipitation

PEAR1 was immunoprecipitated using an anti-PEAR1 antibody and protein A + G beads (Beyotime Biotechnology). IgG were used as a negative control. Cell lysates were the positive control. Protein samples were resolved on a 10% SDS-polyacrylamide gel followed by Coomassie blue staining of the gel. When the experimental group showed specific bands compared with the negative control, the gel was sent for mass spectrometry. After mass spectrometry, immune complexes were blotted with anti-ITGB1 antibody. The same procedure was employed when immunoprecipitation with anti-ITGB1 antibodies was followed by western blotting with anti-PEAR1 antibodies.

### Cell migration assay

MDSCs were seeded in a 12-well plate at 3 × 10^4^ cells/well. Cells were grown to 90% confluence, transfected with VPR/pSPgRNA, VPR/pSPgRNA-P2, or dCas9/pSPgRNA dCas9/pSPgRNA-P3 for 48 h, and scratched with a 200 ml pipette tip to create a linear cell-free zone. The plates were immediately washed three times and added with 1 ml/well of differentiation medium. Defined areas of the cell-free zone were visualized after 0, 24, and 48 h using a phase contrast microscope at a 4× magnification.

### Statistical analysis

Data represent the mean ± SEM from three independent experiments and were analyzed by ANOVA with post-hoc Tukeyʼs tests (SPSS, Inc., Chicago, IL). One-sample t testing was used to assess the statistical significance between groups. Differences were regarded as significant at P < 0.05.

## Supplementary Material

Supplemental Material

## References

[1] Quertermous T, Komuves L, Hart MJ, et al Platelet Endothelial Aggregation Receptor 1 (PEAR1), a novel epidermal growth factor repeat-containing transmembrane receptor, participates in platelet contact-induced activation. J Biol Chem. 2005;280:24680–24689. 15851471 10.1074/jbc.M413411200

[2] Vandenbriele C, Kauskot A, Vandersmissen I, et al Platelet endothelial aggregation receptor-1: A novel modifier of neoangiogenesis. Cardiovasc Res. 2015. 10.1093/cvr/cvv193 26156496

[3] Vandenbriele C, Sun Y, Criel M, et al Dextran sulfate triggers platelet aggregation via direct activation of PEAR1. Platelets. 2016;27:365–372. 10.3109/09537104.2015.1111321 26619766

[4] Dang RP, Schafer JM, Scheib JL, et al The adaptor protein GULP promotes Jedi-1-mediated phagocytosis through a clathrin-dependent mechanism. Mol Biol Cell. 2014;25:1925–1936. 24743597 10.1091/mbc.E13-11-0658 PMC4055271

[5] Krivtsov AV, Rozov FN, Zinovyeva MV, et al Jedi - A novel transmembrane protein expressed in early hematopoietic cells. J Cell Biochem. 2007;101:767–784. 17226770 10.1002/jcb.21232

[6] Geiger B, Spatz JP, Bershadsky AD. Environmental sensing through focal adhesions. Nat Rev Mol Cell Biol. 2009. 10.1038/nrm2593 19197329

[7] Sulzmaier FJ, Jean C, Schlaepfer DD FAK in cancer: mechanistic findings and clinical applications. Nat Rev Cancer. 2014;14:598–610. 25098269 10.1038/nrc3792 PMC4365862

[8] Schaller MD Cellular functions of FAK kinases: insight into molecular mechanisms and novel functions. J Cell Sci. 2010;123:1007–1013. 20332118 10.1242/jcs.045112

[9] Aguilar-Solis ED, Lee-Rivera I, Álvarez-Arce A, et al FAK phosphorylation plays a central role in thrombin-induced RPE cell migration. Cell Signal. 2017;36:56–66. 10.1016/j.cellsig.2017.04.016 28445805

[10] Yuan C, Gou X, Deng J, et al FAK and BMP-9 synergistically trigger osteogenic differentiation and bone formation of adipose derived stem cells through enhancing Wnt-β-catenin signaling. Biomed Pharmacother. 2018;105:753–757. 10.1016/j.biopha.2018.04.185 29909342

[11] Wickström SA, Fässler R Regulation of membrane traffic by integrin signaling. Trends Cell Biol. 2011;21(5):266–73. 10.1016/j.tcb.2011.02.003 21440440

[12] Andreani V, Grosschedl R, Pandey A, et al Cochaperone Mzb1 is a key effector of Blimp1 in plasma cell differentiation and β1-integrin function. Proc Natl Acad Sci. 2018. 10.1073/pnas.1809739115 PMC6187189 30257949

[13] Lu M, Zhuang X, Tang K, et al Intrinsic surface effects of tantalum and titanium on integrin α5β1/ERK1/2 pathway-mediated osteogenic differentiation in rat bone mesenchymal stromal cells. Cell Physiol Biochem. 2018;51:589–609. 30458456 10.1159/000495280

[14] Bae GU, Gaio U, Yang YJ, et al Regulation of myoblast motility and fusion by the CXCR4-associated sialomucin, CD164. J Biol Chem. 2008. 10.1074/jbc.M706730200 PMC2276390 18227060

[15] Cornelison DDW, Filla MS, Stanley HM, et al Syndecan-3 and syndecan-4 specifically mark skeletal muscle satellite cells and are implicated in satellite cell maintenance and muscle regeneration. Dev Biol. 2001;239(1):79–94. 10.1006/dbio.2001.0416 11784020

[16] Cui YF, Yan YQ, Liu D, et al Platelet endothelial aggregation receptor-1 (PEAR1) is involved in C2C12 myoblast differentiation. Exp Cell Res. 2018;366(2):199–204. 10.1016/j.yexcr.2018.03.027 29577896

[17] Tong HL, Yin HY, Zhang WW, et al Transcriptional profiling of bovine muscle-derived satellite cells during differentiation in vitro by high throughput RNA sequencing. Cell Mol Biol Lett. 2015. 10.1515/cmble-2015-0019 26208385

[18] Birchmeier C, Brohmann H Genes that control the development of migrating muscle precursor cells. Curr Opin Cell Biol. 2000;12(6):725–30. 10.1016/s0955-0674(00)00159-9 11063939

[19] Mylona E, Jones KA, Mills ST, et al CD44 regulates myoblast migration and differentiation. J Cell Physiol. 2006. 10.1002/jcp.20724 16906571

[20] Parsons JT Focal adhesion kinase: the first ten years. J Cell Sci. 2003. 10.1242/jcs.00373 12640026

[21] Bagi CM, Roberts GW, Andresen CJ Dual focal adhesion kinase/Pyk2 inhibitor has positive effects on bone tumors: implications for bone metastases. Cancer. 2008;112:2313–2321. 18348298 10.1002/cncr.23429

[22] Shafaq-Zadah M, Gomes-Santos CS, Bardin S, et al Persistent cell migration and adhesion rely on retrograde transport of β 1 integrin. Nat Cell Biol. 2016. 10.1038/ncb3287 26641717

[23] Zhao XK, Cheng Y, Liang Cheng M, et al Focal adhesion kinase regulates fibroblast migration via integrin beta-1 and plays a central role in fibrosis. Sci Rep. 2016;6:19276. 10.1038/srep19276 PMC4725867 26763945

[24] Cheung TH, Quach NL, Charville GW, et al Maintenance of muscle stem-cell quiescence by microRNA-489. Nature. 2012. 10.1038/nature10834 PMC3292200 22358842

[25] Wang Y, Zhang ZT, Seo SO, et al Gene transcription repression in Clostridium beijerinckii using CRISPR-dCas9. Biotechnol Bioeng. 2016;113(12):2739–2743. 10.1002/bit.26020 27240718

